# A Clinical-Operational Framework for Responsible Artificial Intelligence Use in Infection Risk Management Among Immunosuppressed Patients: A Technical Report

**DOI:** 10.7759/cureus.110205

**Published:** 2026-06-03

**Authors:** Alejandro Rodriguez Bobadilla, Sergio Alonso Bobadilla Ocampo, Ingrid Carolina Bobadilla Ocampo

**Affiliations:** 1 Infectious Disease, Universidad del Rosario, Bogota, COL; 2 General Practice, Fundación Universitaria Juan N. Corpas, Bogotá, COL; 3 Medicine, Fundación Universitaria Juan N. Corpas, Bogotá, COL

**Keywords:** antimicrobial stewardship, artificial intelligence, bloodstream infections, clinical decision support, clinical governance, immunocompromised patients, latent tuberculosis infection, machine learning, sepsis

## Abstract

Immunosuppressed patients carry a disproportionate infectious risk driven by underlying disease, biologic or immunomodulatory therapies, glucocorticoid exposure, comorbidities, latent infections, antimicrobial resistance, and fragmented follow-up. Artificial intelligence may support infection risk management by integrating clinical, microbiological, pharmacological, and operational data; however, it should not be implemented as an autonomous solution or a substitute for clinical judgment.

This technical report proposes a clinical-operational framework for the responsible use of artificial intelligence in infection risk management among immunosuppressed patients. The framework includes seven domains: baseline infection risk stratification, screening and follow-up of latent infections, alerts for infectious deterioration, support for rational antimicrobial use, antimicrobial resistance surveillance, traceable clinical documentation, and continuous performance auditing. To reduce risks related to bias, false reassurance, alert fatigue, limited transportability, performance degradation, and excessive reliance on model outputs, the framework emphasizes local validation, human oversight, proportional explainability, institutional governance, longitudinal monitoring, adverse event review, and formal updating mechanisms.

The objective is not to claim that artificial intelligence independently reduces infections or improves outcomes, but to define minimum conditions for its use as a supervised support tool within integrated programs for prevention, antimicrobial stewardship, patient safety, and risk management. This approach may be useful when prevention of latent tuberculosis, bacteremia, sepsis, opportunistic infections, and antimicrobial resistance requires coordinated clinical, microbiological, pharmacological, technological, quality, and audit processes.

## Introduction

Immunosuppressed patients represent a highly complex population for infection risk management. Exposure to biologic therapies, immunomodulators, glucocorticoids, chemotherapy, or transplantation, together with autoimmune diseases, hematologic malignancies, and relevant comorbidities, can alter the clinical presentation of infection, increase the risk of severe infection, bacteremia, and sepsis, and complicate the interpretation of clinical, microbiological, and pharmacological markers [1]. In this context, prevention requires anticipating risk trajectories, ensuring screening for latent infections, optimizing antimicrobial use, detecting early clinical deterioration, and maintaining longitudinal traceability of clinical decisions.

For the purposes of this report, artificial intelligence refers to computational systems designed to support tasks that usually require human reasoning, such as pattern recognition, risk estimation, prioritization, and decision support [2]. Machine learning is a subset of artificial intelligence in which algorithms learn patterns from data rather than relying exclusively on predefined rules [2]. In supervised learning, models are trained using labeled examples, such as patients with or without sepsis, bloodstream infection, antimicrobial resistance, or another predefined outcome. Common supervised learning approaches include regression-based models, decision trees, random forests, gradient boosting methods, neural networks, and deep learning architectures [2-4]. In clinical practice, these methods should be interpreted as tools that generate probabilistic support signals, not as autonomous diagnostic or therapeutic authorities [5,6].

Artificial intelligence and machine learning can support this process by integrating clinical, demographic, pharmacological, microbiological, molecular, and operational data that exceed the capacity of real-time manual review. In infectious diseases, this capability is particularly relevant because of diagnostic uncertainty, delays inherent to some microbiological results, clinical workload pressure, the need for antimicrobial stewardship, and antimicrobial resistance surveillance [2-4]. However, the technical potential of these models does not automatically translate into clinical utility. In immunosuppressed patients, biological vulnerability and atypical presentations of infection require local validation, safe integration into clinical workflows, and human oversight. Studies in hematopoietic stem cell transplant recipients show that models designed for immunocompromised populations may outperform general clinical tools, but also that performance depends on the population, selected outcome, institutional context, and real-world timing of clinical decision-making [1].

The clinical artificial intelligence literature has highlighted a persistent gap between retrospective model performance and demonstrable impact on patient care. DECIDE-AI emphasizes that artificial intelligence-driven decision support systems should be evaluated as complex interventions, considering safety, early clinical utility, human factors, and workflow integration. FUTURE-AI further proposes that artificial intelligence in healthcare should be fair, universal, traceable, usable, robust, and explainable throughout its life cycle [5,6].

Prudence is critical in infectious diseases because algorithmic errors may lead to delayed antimicrobial therapy, unnecessary antibiotic exposure, alert fatigue, false reassurance, or decisions inconsistent with clinical judgment. External validation of the Epic Sepsis Model showed poor discrimination and calibration, low sensitivity, and a substantial alert burden, demonstrating that broad adoption of predictive models does not guarantee safety or clinical value without independent evaluation and continuous monitoring [7]. In addition, models may degrade over time because of changes in patient populations, clinical practice, information systems, and data quality; therefore, they require longitudinal surveillance, controlled updating, institutional quality improvement processes, and responsible governance [8,9].

## Technical report

Scope, target population, and supported decisions

This technical report proposes a clinical-operational framework to guide the responsible use of artificial intelligence in infection risk management among immunosuppressed patients. The framework is designed to support, not replace, clinical decision-making in settings where risk depends on underlying disease, intensity of immunosuppression, pharmacological exposure, comorbidities, latent infections, prior microbiology, antimicrobial resistance, clinical trajectory, and continuity of follow-up.

The target population includes patients with pharmacological immunosuppression, immunosuppression secondary to underlying disease, or immunosuppression associated with complex therapeutic procedures, including patients with autoimmune diseases treated with biologic or immunomodulatory therapies, patients receiving systemic glucocorticoids, transplant recipients, patients with hematologic malignancies, patients receiving chemotherapy, and people with advanced HIV infection. Before implementing the tool, the institution must define which decision will be supported, where in the care pathway the tool will be activated, what action is expected, and who will be responsible for interpreting it.

Functional architecture of the framework

The framework is organized into three layers. The data layer integrates clinical, demographic, pharmacological, microbiological, radiological, laboratory, and operational information. The decision layer transforms these data into supportive signals, such as risk stratification, clinical alerts, follow-up prioritization, identification of screening gaps, antimicrobial review, or activation of institutional pathways. The governance layer defines who authorizes the tool, how it is validated, how it is monitored, how its outputs are documented, and under what conditions it should be modified, suspended, or withdrawn.

For operational use, the framework should explicitly distinguish four elements: input data, processed signals, model outputs, and human clinical action. Input data include variables extracted from the electronic health record, laboratory systems, microbiology reports, pharmacy records, imaging reports, and operational follow-up processes. Processed signals are intermediate risk patterns generated from these inputs, such as incomplete screening pathways, abnormal serial laboratory trends, prior resistant isolates, antimicrobial discordance, or deterioration trajectories. Model outputs are the visible alerts, risk tiers, prioritization flags, or documentation prompts delivered to the clinical team. Human clinical action refers to the documented decision to accept, modify, reject, or contextualize the output according to the patient’s condition and institutional protocols.

Missingness should not be interpreted as absence of risk. Missing or incomplete data should be explicitly labeled according to their operational meaning, such as unavailable, pending, not performed, not applicable, outdated, or not interoperable. When critical variables are missing, the output should communicate uncertainty, restrict confidence in the generated signal, or trigger human review rather than provide false reassurance. Critical missingness should be auditable, especially when it involves latent infection screening, microbiology results, antimicrobial exposure, renal function, allergies, current immunosuppression, or recent clinical deterioration.

**Figure 1:**
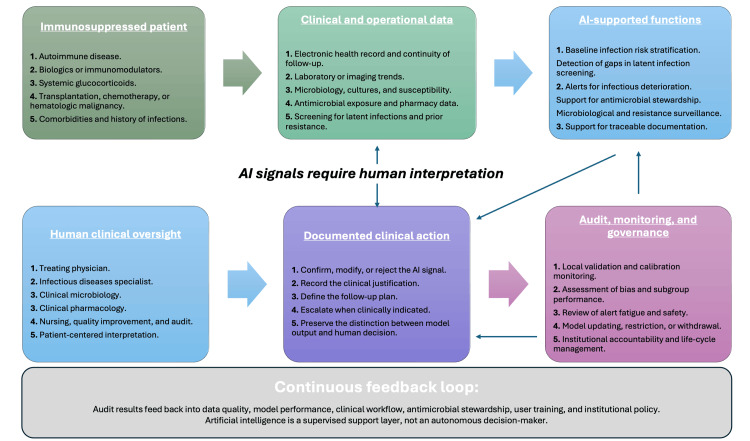
Clinical-operational workflow for the responsible use of artificial intelligence in infection risk management among immunosuppressed patients The workflow illustrates artificial intelligence as a supervised support layer, not as an autonomous decision-maker. Clinical and operational data are transformed into risk signals, alerts, antimicrobial stewardship support, and documentation support. Human teams retain responsibility for clinical interpretation and action, while institutional governance provides validation, monitoring, bias assessment, audit, life-cycle management, and criteria to modify, restrict, or withdraw the model. Figure created using Microsoft PowerPoint

Implementation requires sufficient interoperability with the medical record, laboratory, microbiology, pharmacy, and quality processes. When interoperability or data quality is incomplete, clinical use should be restricted and outputs should be interpreted with caution. Figure 1 summarizes the proposed clinical-operational workflow, from identification of the immunosuppressed patient and data integration to the generation of supportive signals, human oversight, traceable documentation, continuous audit, and institutional governance.

The clinical-operational domains of the framework translate the general workflow into concrete institutional functions, each linked to a required human action and an auditable indicator. These domains are presented in Table 1.

**Table 1:** Clinical-operational domains for the responsible use of artificial intelligence in infection risk management among immunosuppressed patients

Domain	Operational purpose	Function supported by artificial intelligence	Required human action	Auditable indicator
Baseline infection risk stratification	Identify patients with a higher probability of infectious complications before the clinical event	Integration of comorbidities, immunosuppression, prior microbiology, antimicrobial exposure, and care trajectory	Priority clinical review, medication reconciliation, and definition of the follow-up plan	Proportion of patients stratified before initiating or escalating immunosuppression
Screening for latent infections	Detect gaps in preventive pathways before or during immunosuppression	Identification of missing tests, pending results, documentation discrepancies, and need for follow-up	Clinical confirmation, exclusion of active disease, and documented preventive decision	Proportion of patients with a complete and traceable screening pathway
Alerts for infectious deterioration	Prioritize patients with possible severe infection, bacteremia, sepsis, or opportunistic infection	Generation of signals based on physiological changes, serial laboratory data, microbiology, and therapeutic context	Immediate or priority clinical evaluation according to severity and context	Time from alert to documented evaluation
Optimization of antimicrobial use	Support review of antimicrobial initiation, continuation, de-escalation, duplication, toxicity, or discontinuation	Detection of discordance between antimicrobial therapy, culture, focus, renal function, allergies, and prior exposure	Confirmation by the treating team, clinical pharmacy, or infectious diseases team	Proportion of antimicrobial alerts reviewed and documented
Microbiological surveillance and antimicrobial resistance	Integrate cultures, susceptibility results, previous isolates, and institutional resistance patterns	Identification of risk for resistant microorganisms, institutional trends, and diagnostic gaps	Interpretation by microbiology, infectious diseases, or the infection committee	Periodic updating of microbiological profiles and resistance reports
Traceable clinical documentation	Distinguish input data, model output, and human decision	Structured recording of alerts, recommendations, acceptance, modification, or rejection	Documented clinical justification when the decision differs from the generated signal	Proportion of interactions with complete and retrievable documentation
Institutional governance	Ensure validation, monitoring, safety, equity, and life-cycle control	Model registry, risk assessment, performance monitoring, and detection of degradation	Approval, follow-up, updating, or withdrawal by a multidisciplinary committee	Existence of an active model inventory and documented periodic review

Clinical-operational domains of the framework

The framework includes seven interdependent domains. Baseline infection risk stratification aims to identify patients with a higher probability of complications, unresolved latent infections, colonization with resistant microorganisms, prior antimicrobial exposure, relevant comorbidities, or follow-up gaps. This classification should integrate host factors, underlying disease, type and intensity of immunosuppression, prior microbiology, local resistance patterns, and operational variables. The framework does not propose a universal high-risk scale because infectious risk in immunosuppressed patients is context-dependent. High risk should be defined as a locally validated risk tier linked to a specific clinical objective, such as prioritizing completion of latent infection screening, infectious diseases review, antimicrobial review, closer follow-up, or early clinical evaluation. Institutions should define thresholds according to local epidemiology, available data, patient population, therapeutic context, and response capacity. A high-risk result should trigger priority clinical review, medication reconciliation, screening verification, and definition of follow-up; a low-risk result should not rule out infection when discordant clinical data or physiological deterioration are present.

Screening and follow-up of latent infections should detect missing tests, pending results, documentation discrepancies, and failures in preventive continuity. For latent tuberculosis infection, the tool should verify immunological tests, clinical evaluation, exclusion of active tuberculosis when appropriate, chest imaging or another indicated study, therapeutic decision, prophylaxis if applicable, and follow-up of tolerance, adherence, and closure of the process. Its function is not to diagnose or block treatments autonomously, but to alert the responsible team when the preventive pathway is incomplete.

Alerts for infectious deterioration should consider that infection in immunosuppressed patients may present without fever, with attenuated biomarkers, nonspecific symptoms, or progressive functional decline. Therefore, alerts should integrate physiological variables, serial laboratory data, microbiology, therapeutic context, and individual trajectory. Each alert should answer a specific clinical question and have a recipient, time frame, urgency level, and expected action. Repetitive, nonspecific, or non-actionable alerts should be considered design failures.

Support for optimization of antimicrobial use should organize information on suspected source, severity, renal function, allergies, prior antimicrobials, historical cultures, colonization with resistant microorganisms, local susceptibility patterns, drug interactions, and active immunosuppression. Its function should be limited to review signals, such as possible de-escalation, duplication, discordance between culture and treatment, toxicity risk, or need for infectious diseases evaluation. In immunosuppressed patients, antimicrobial optimization should balance the harm of unnecessary treatment against the harm of insufficient or delayed treatment.

Microbiological and antimicrobial resistance surveillance should integrate cultures, susceptibility results, previous isolates, cumulative antimicrobial exposure, and institutional resistance patterns. This information may modify the pretest probability of infection caused by resistant or unusual microorganisms and support population-level surveillance, trend identification, prioritization of preventive interventions, and evaluation of antimicrobial consumption. Aggregate results should not be automatically extrapolated to individual decisions without clinical and microbiological interpretation.

Traceable clinical documentation should allow reconstruction of the user, relevant available data, generated output, recommended action, clinical decision adopted, and justification when the decision differs from the suggestion. Documentation should distinguish input data, model output, and human decisions, including model version when there are changes in variables, thresholds, architecture, interface, data source, or target population.

Institutional governance should ensure that every artificial intelligence tool applied to infection management among immunosuppressed patients is registered in an institutional model inventory, including purpose, target population, data used, clinical and technical leads, validation status, risk level, known limitations, performance metrics, monitoring plan, and suspension criteria. Governance should be multidisciplinary and include infectious diseases, microbiology, pharmacy, nursing, specialties using immunosuppression, epidemiology, patient safety, quality improvement, clinical audit, technology, data protection, and institutional leadership. The main implementation risks and their minimum control mechanisms are presented in Table 2.

**Table 2:** Risk-control matrix for the responsible implementation of artificial intelligence in immunosuppressed patients

Implementation risk	Potential clinical or operational consequence	Required mitigation	Responsible team	Monitoring indicator
Incomplete or low-quality data	Erroneous alerts, false reassurance, or inappropriate prioritization	Data quality audit, completeness rules, and flagging of missing data	Technology, quality improvement, laboratory, clinical team	Proportion of outputs with missing critical data
Limited model transportability	Inferior performance when applied to a different population or institution	Local validation before clinical use and restriction to the approved purpose	Governance committee, data science, and clinical leaders	Local performance against predefined thresholds
Temporal performance degradation	Progressive loss of sensitivity, specificity, or calibration	Longitudinal monitoring and formal criteria for updating or suspension	Data science, quality improvement, and patient safety	Temporal trend in discrimination, calibration, and utility
Alert fatigue	Omission of relevant signals and reduced user adherence	Actionable thresholds, severity-based prioritization, and periodic review of ignored alerts	Clinical team, quality improvement, and technology	Proportion of non-actioned or repetitive alerts
Bias against subgroups	Inequity in prioritization, diagnosis, or follow-up	Subgroup evaluation and model correction or restriction if differential performance exists	Governance, ethics, quality improvement, and data science	Performance metrics by clinical and sociodemographic subgroup
Excessive reliance on the model	Uncritical automation and displacement of clinical judgment	Training, explanation of limitations, and mandatory human confirmation	Clinical lead, medical education, and patient safety	Proportion of decisions documented without human review
Lack of operational explainability	Low trust, system rejection, or inappropriate use	Presentation of contributing factors, uncertainty, and suggested action	Data science, technology, and clinical users	Usability surveys and discrepancy review
Absence of traceability	Inability to audit decisions, incidents, or version changes	Structured recording of input data, output, human decision, and model version	Technology, quality improvement, and clinical audit	Proportion of interactions with complete traceability

Implementation, oversight, and audit

Implementation should be phased: definition of the clinical-operational problem, target population, supported decision, data sources, and foreseeable risks; retrospective validation and data quality review; silent-mode evaluation without modifying clinical decisions; supervised pilot testing with trained users; and gradual scaling conditioned on acceptable local performance, operational utility, and absence of safety signals.

Deployment should not depend only on statistical metrics. The institution should assess timeliness of the alert, interpretability, cognitive burden, workflow integration, user acceptance, equity, performance stability, and real response capacity. Pause or withdrawal criteria should exist for performance degradation, non-actionable alerts, subgroup bias, interoperability failures, data security concerns, or use outside the approved purpose.

Human oversight is a structural requirement. The responsible professional should understand the tool’s purpose, limitations, main variables, operational meaning of its outputs, and expected action. The tool should communicate uncertainty in a clinically understandable way, identifying contributing factors, missing data, and conditions under which the output should not be used. Clinicians should be able to accept, modify, or reject the signal with reasoned justification.

Audit should evaluate model performance, operational impact, clinical safety, equity, and documentation quality. Performance should include discrimination, calibration, sensitivity, specificity, predictive values, temporal stability, and subgroup performance. Continuous improvement should distinguish operational adjustments from model updates; changes in variables, thresholds, architecture, or target population require new technical evaluation, version documentation, and subsequent monitoring.

Expected operational result

The expected result is not to demonstrate direct clinical effectiveness, but to establish a responsible pathway for artificial intelligence to support infection management programs for immunosuppressed patients. Adequate implementation should help identify screening gaps, prioritize follow-up for higher-risk patients, improve traceability of decisions, support antimicrobial review, strengthen microbiological surveillance, and generate auditable indicators for quality and patient safety.

The framework should be interpreted as a risk management infrastructure. Its value depends on data quality, institutional digital maturity, participation of clinical teams, capacity to respond to alerts, governance of the model life cycle, and continuous review of results. Without these elements, artificial intelligence may amplify existing errors or produce signals that do not translate into better care.

## Discussion

The main contribution of this technical report is to propose a clinical-operational framework for applying principles of responsible artificial intelligence to infection risk management among immunosuppressed patients. The literature on machine learning in medicine, antimicrobial learning systems, and antimicrobial resistance prediction shows that these systems can integrate clinical, microbiological, pharmacological, and operational data that exceed the capacity of real-time human review, but that their clinical value depends on validation, transparency, explainability, local oversight, and safe integration into clinical workflows [2-4]. In immunosuppressed patients, this need is particularly relevant because of atypical infection presentations, complex pharmacological exposure, prior microbiology, antimicrobial resistance, and the narrow margin for diagnostic or therapeutic errors. Evidence in hematopoietic stem cell transplant recipients supports the potential value of models tailored to immunocompromised populations, but also confirms that performance depends on the population, outcome, institutional context, and real-world timing of clinical decision-making [1].

A central contribution of the framework is to shift the emphasis from the isolated model to the complete clinical workflow. DECIDE-AI and FUTURE-AI agree that artificial intelligence-based decision support systems should be evaluated as complex interventions, incorporating safety, human factors, early clinical utility, traceability, usability, robustness, explainability, and life-cycle monitoring [5,6]. In this report, these principles are translated into concrete operational domains: risk stratification, screening for latent infections, alerts for infectious deterioration, antimicrobial review, microbiological surveillance, traceable documentation, and institutional governance.

Recent evidence in sepsis and infection illustrates both the potential and the risks of clinical artificial intelligence. External validation of the Epic Sepsis Model showed poor discrimination and calibration, low sensitivity, and a substantial alert burden, indicating that broad adoption of predictive models does not guarantee safety or clinical value [7]. In addition, models may degrade because of changes in patient mix, local epidemiology, clinical practice, information systems, and data quality; therefore, responsible deployment requires local validation, calibration surveillance, alert fatigue analysis, longitudinal monitoring, an institutional model inventory, and explicit criteria for modification, restriction, or withdrawal [8,9].

In contrast, some experiences show that artificial intelligence may add value when properly integrated into clinical workflows and maintained under human oversight. TREWS was associated with better outcomes in sepsis when clinicians confirmed the alert in a timely manner; TriVerity showed diagnostic and prognostic potential in acute infection through mRNA expression and machine learning; longitudinal deep learning models have shown potential utility for identifying bloodstream infections before blood culture results are available; and the AI Clinician illustrated the use of reinforcement learning to model therapeutic strategies in critical sepsis [10-13]. These studies do not imply that artificial intelligence tools are interchangeable or that model performance alone ensures clinical benefit. Rather, they show that clinical value depends on the combination of model, context, data, therapeutic timing, human response, and institutional audit capacity.

Representative examples of artificial intelligence and machine learning applications relevant to infection risk management are summarized in Table 3. These examples are not presented as interchangeable tools, but as evidence that different model types may support different infection-related tasks depending on the population, outcome, data source, timing, and clinical workflow.

**Table 3:** Representative artificial intelligence and machine learning applications relevant to infection risk management ML: Machine learning

Clinical area or outcome	Representative AI/ML approach	Intended use	Representative reference
Bacterial sepsis in immunocompromised stem cell transplant recipients	Supervised machine learning model	Estimation of bacterial sepsis risk in an immunocompromised population	Lind et al. [1]
Sepsis prediction in hospitalized patients	Proprietary predictive model	Early warning for possible sepsis	Wong et al. [7]
Sepsis early warning	Machine learning-based early warning system	Timely clinician-confirmed sepsis alert and workflow activation	Adams et al. [10]
Acute infection and sepsis	Host mRNA expression combined with machine learning	Diagnostic and prognostic support in acute infection	Liesenfeld et al. [11]
Bacterial bloodstream infection	Time-series deep learning using routinely collected clinical data	Earlier identification of bloodstream infection before culture confirmation	Ming et al. [12]
Antimicrobial resistance	Machine learning applied to microbiological, genomic, or clinical data	Prediction or surveillance support for antimicrobial resistance	Kim et al. [4]
Sepsis treatment strategies	Reinforcement learning	Modeling therapeutic strategies in critical illness	Komorowski et al. [13]

The examples illustrate heterogeneous applications of artificial intelligence and machine learning in infection-related contexts. They should not be interpreted as directly comparable tools because they differ in population, data source, target outcome, timing, validation status, and intended clinical use.

In antimicrobial stewardship, the framework avoids interpreting artificial intelligence as a simple mechanism to reduce antibiotic use. In immunosuppressed patients, the objective is to balance opposing risks: unnecessary antimicrobial exposure, toxicity, and selection of resistance versus delayed therapy, insufficient coverage, or failure to recognize severe infection. The same logic applies to latent tuberculosis and other preventable infections before immunosuppression, where the value of the system lies in detecting operational gaps, pending results, incomplete preventive pathways, or lack of follow-up, not in replacing clinical reasoning.

This technical report does not evaluate a specific algorithm or measure clinical outcomes of its own. Its contribution is to propose a clinical-operational framework to guide responsible implementation in a high-risk population. Its application requires local adaptation according to digital maturity, data quality, interoperability, availability of clinical microbiology, human resources, and institutional response capacity. The next step should be to convert this framework into an evaluable protocol, beginning with retrospective validation, silent-mode evaluation, supervised piloting, and safety monitoring before moving toward clinical impact studies.

## Conclusions

Artificial intelligence can strengthen infection risk management among immunosuppressed patients when implemented as a supervised clinical support tool, integrated into defined care workflows and supported by local validation, traceability, continuous monitoring, and institutional governance. Its value depends less on isolated prediction than on its ability to support critical processes such as risk stratification, screening for latent infections, microbiological surveillance, antimicrobial stewardship, clinical documentation, and safety auditing.

The proposed framework provides an operational structure to guide responsible, stepwise, and measurable implementation in populations with high vulnerability to infection. Its application should remain centered on human clinical judgment and should initially be assessed through indicators of feasibility, safety, acceptability, documentation quality, and operational response before advancing to the evaluation of clinical outcomes.
